# Direct Visualization of the Evolution of a Single‐Atomic Cobalt Catalyst from Melting Nanoparticles with Carbon Dissolution

**DOI:** 10.1002/advs.202200592

**Published:** 2022-05-04

**Authors:** Luyao Zhang, Yanyan Li, Lei Zhang, Kun Wang, Yingbo Li, Lei Wang, Xinyu Zhang, Feng Yang, Zhiping Zheng

**Affiliations:** ^1^ Department of Chemistry Guangdong Provincial Key Laboratory of Catalysis Guangdong Provincial Key Laboratory of Energy Materials for Electric Power Key Laboratory of Energy Conversion and Storage Technologies (Ministry of Education) Southern University of Science and Technology Shenzhen 518055 China

**Keywords:** carbon dissolution, environmental transmission electron microscope, molten nanoparticles, single‐atom catalyst, structure evolution

## Abstract

Transition metal single‐atom catalysts (SACs) are of immense interest, but how exactly they are evolved upon pyrolysis of the corresponding precursors remains unclear as transition metal ions in the complex precursor undergo a series of morphological changes accompanied with changes in oxidation state as a result of the interactions with the carbon support. Herein, the authors record the complete evolution process of Co SAC during the pyrolysis a Co/Zn‐containing zeolitic imidazolate framework. Aberration‐corrected environmental TEM coupled with in‐situ EELS is used for direct visualization of the evolution process at 200–1000 °C. Dissolution of carbon into the nanoparticles of Co is found to be key to modulating the wetting behavior of nanoparticles on the carbon support; melting of Co nanoparticles and their motion within the zeolitic architecture leads to the etching of the framework structure, yielding porous C/N support onto which Co‐single atoms reside. This uniquely structured Co SAC is found to be effective for the oxidation of a series of aromatic alkanes to produce selective ketones among other possible products. The carbon dissolution and melting/sublimation‐driven structural dynamics of transition metal revealed here will expand the methodology in synthesizing SACs and other high‐temperature processes.

## Graphical abstract

The origins of Co single atom catalyst anchored on a porous CN*_x_* support is unveiled by in‐situ environmental TEM coupled with EELS, which is associated with carbon dissolving into Co induced wetting of the nanoparticles on the carbon support, melting of the Co nanoparticles, and their motion within the zeolitic architecture leads to the etching of the framework structure.

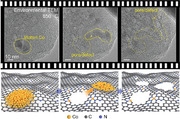

## 1 Introduction

Single‐atom catalysts (SACs)^[^ ^1^, ^2^ ^]^ are of tremendous recent research interest because of the potential maximized utilization of metal atoms, tailorable and uniform metal active sites, unique reaction pathway,^[^ ^3^, ^4^, ^5^, ^6^, ^7^, ^8^, ^9^ ^]^ and their diverse applications, in particular for reactions of environmental and energy significance.^[^ ^9^, ^10^, ^11^, ^12^, ^13^, ^14^, ^15^, ^16^, ^17^, ^18^, ^19^ ^]^ Among the various approaches developed for the preparation of SACs,^[^ ^20^, ^21^, ^22^, ^23^, ^24^, ^25^, ^26^, ^27^, ^28^, ^29^ ^]^ pyrolysis of metal–organic framework^[^ ^30^, ^31^, ^32^, ^33^, ^34^, ^35^ ^]^ and other forms of metal complex,^[^ ^13^, ^36^, ^37^, ^38^, ^39^ ^]^ resulting in single‐atom metal sites loaded on porous carbon‐based support and a tailored metal‐coordination environment, has received arguably the most attention. The ability to capture the dynamics of single‐atom evolution during pyrolysis is particularly important for understanding the mechanism of SAC origin.^[^ ^40^ ^]^ Most of these studies have concentrated on SACs of noble metals. Pioneer works have been reported on the transformation of nanosized noble metals (Pt, Au, Ag, Ir) to SACs using environmental transmission electron microscope (ETEM),^[^ ^9^, ^32^, ^41^, ^42^, ^43^, ^44^ ^]^ though much less is known about the transition metal single atoms and their interaction with support.^[^ ^45^, ^46^, ^47^ ^]^ Great progress notwithstanding, the process of metal atomization remains unclear, so does the formation of the porous carbon support.

As compared with noble metals, the formation of SACs of other transition metals such as Fe, Co, and Ni is even more complicated. This complicity arises from two specific properties of these earth‐abundant metal elements. First, these non‐noble transition metals have a lower melting point (1200–1500 °C) than that of noble metal (e.g., Pt, Ru, Rh) (1700–2400 °C);^[^ ^48^ ^]^ nanoparticles of these elements are more likely to melt than their noble‐metal counterparts at the pyrolysis temperature (normally >600 °C). Second, it is generally accepted that carbon has a larger affinity for transition metal, leading to stronger adhesion (interactions) between these metals and a carbon‐support than the noble metals.^[^ ^49^, ^50^, ^51^, ^52^ ^]^ Therefore, dissolution of carbon into transition metal nanoparticles may occur, especially near the surface of a nanoparticle.^[^ ^53^, ^54^, ^55^, ^56^ ^]^ Computational studies have predicted that the wetting properties of a nanoparticle on the surface of a support are profoundly influenced by the amount of carbon inside a transition metal nanoparticle.^[^ ^49^, ^57^ ^]^ As such, it would be possible to modulate the energetics at the nanoparticle‐carbon support interface, so as to fine‐tune the properties of the resulting SACs. However, the complex interactions between the metal atoms and the carbon support under the conditions typically used for the preparation of SACs have never been revealed experimentally.

The macroscale integral spectroscopic methods such as Mössbauer,^[^ ^58^, ^59^ ^]^ infrared,^[^ ^60^ ^]^ and synchrotron X‐ray absorption spectroscopy (XAS)^[^ ^61^, ^62^, ^63^ ^]^ provide the electronic‐level insight into SAC mechanism under a homogeneous modeling assumption. However, given practical systems with a structural diversity, where single atoms, clusters, and particles coexist, the spectroscopic methods unfortunately become weak to reflect the localized structure. In‐situ ETEM, capable of real‐space imaging of atomic structure, has recently emerged as a powerful technique to trace the dynamic evolution of catalysts.^[^ ^64^, ^65^, ^66^, ^67^, ^68^, ^69^, ^70^, ^71^, ^72^, ^73^, ^74^, ^75^, ^76^ ^]^ Moreover, TEM is not chemically sensitive and incapable of differentiating the elements. The reliable way is to combine TEM imaging with electron energy loss spectrum (EELS) simultaneously, thereby allowing the tracking of both structural and chemical evolution.^[^ ^54^, ^77^ ^]^

Using a Co/Zn‐based zeolite imidazolate framework (Co/Zn‐ZIF) as the precursor for pyrolysis, we present here the first direct observation of the evolution of Co single atoms accompanied with the formation of the porous N‐doped carbon support. Combined use of aberration‐corrected ETEM, in‐situ EELS, and synchrotron XAS provided direct visualization and chemical evolution of single Co atoms anchored on the porous N‐doped carbon support derived from the original imidazolate ligand. The unique application of this Co‐SAC for selective oxidation of ethylbenzene to produce acetophenone out of a number of possible products was also demonstrated.

## 2 Results and Discussion

A zeolite imidazolate framework containing both Co and Zn (Co/Zn‐ZIF) was used as precursor for the Co‐based catalysts featuring Co as single atoms, nanoclusters, or nanoparticles. The Co/Zn‐ZIF precursor was prepared by adopting a literature procedure^[^ ^30^ ^]^ and characterized by energy dispersive X‐ray (EDX) elemental mapping and X‐ray diffraction (XRD) (Figure S1, Supporting Information). Upon pyrolysis, Co/Zn‐ZIF is expected to undergo both structural and compositional changes with the transition from a well‐defined metal–organic framework to catalysts featuring different‐sized Co atoms ranging from single atoms to nanocluster, and to nanoparticles conditioned with the pyrolysis temperature. Accompanying the physical state changes are the changes in the composition of the resulting catalysts and the valence states of Co in various forms (e.g., solid, molten, or metastable, pure metal, or metallic compound) produced at different stages of the pyrolysis.

### 2.1 Microstructural Evolution during the Pyrolysis of Co/Zn‐ZIF Monitored by ETEM

**Figure 1:**
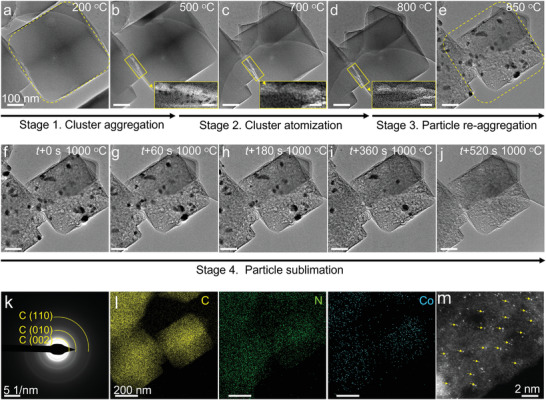
In‐situ ETEM characterization of the ZIF pyrolysis. a–j) Temperature‐sequenced in‐situ TEM images from 200–1000 °C. f–j) Time‐sequenced in‐situ TEM images at 1000 °C. Inset in (b–d): enlarged region showing the nanoparticles and disappearance, scale bars: 20 nm. k–m) SAED pattern (k), EDX elemental mapping (l), and HAADF‐STEM image (m) of single Co atoms on CN*_x_* after 1000 °C‐ETEM. The arrows are meant to help with observation of Co single atoms.

**Figure** **1** depicts the 4‐stage microstructural evolution upon pyrolysis of Co/Zn‐ZIF from 200 to 1000 °C. Although pyrolysis up to 200 °C did not generate any noticeable morphological changes to the Co/Zn‐ZIF structure (Figure 1a and Figure S2, Supporting Information), a close‐up view of the ETEM image of the sample pyrolyzed at 500 °C reveals the formation of tiny clusters and further atomically dispersed at 700–800 °C (Figure 1a–d, Stages 1 and 2). We also carefully analyzed the process at 500–800 °C by in‐situ high‐angle‐annular dark field scanning TEM (HAADF‐STEM) coupled with EELS elemental mapping, showing the similar evolution that the clusters digested within the support (Figures S3 and S4, Supporting Information). Increasing the temperature to 850 °C led to the growth of this dispersed cobalt species into larger nanoparticles, accompanied with the shrinkage of the ZIF framework into a porous structure (Figure 1e, marked by dashed framework, Stage 3). Further, the increase of the temperature to 1000 °C led to the sublimation and eventual disappearance of the nanoparticles after heating at this temperature for 520 s; consistent with the ETEM results is the absence of diffraction peaks of nanoparticles (Figure 1f–k, Stage 4, and Video S1, Supporting Information) in the selected‐area electron diffraction (SAED). Instead, the STEM‐EDX elemental mapping and aberration‐corrected HAADF‐STEM images collected from the same region show that single atoms of Co distributed on N‐doped carbon support (Figures 1l and 1m, and Figures S5 and S6, Supporting Information).

### 2.2 Chemical Evolution during the Pyrolysis of Co/Zn‐ZIF Monitored by In‐Situ EELS

As TEM is not chemically sensitive and incapable of differentiating the elements, we resort to EELS to track the compositional changes as the pyrolysis progressed as the energy loss near the edge structure is sensitively dependent on the coordination environment of the element.

**Figure 2:**
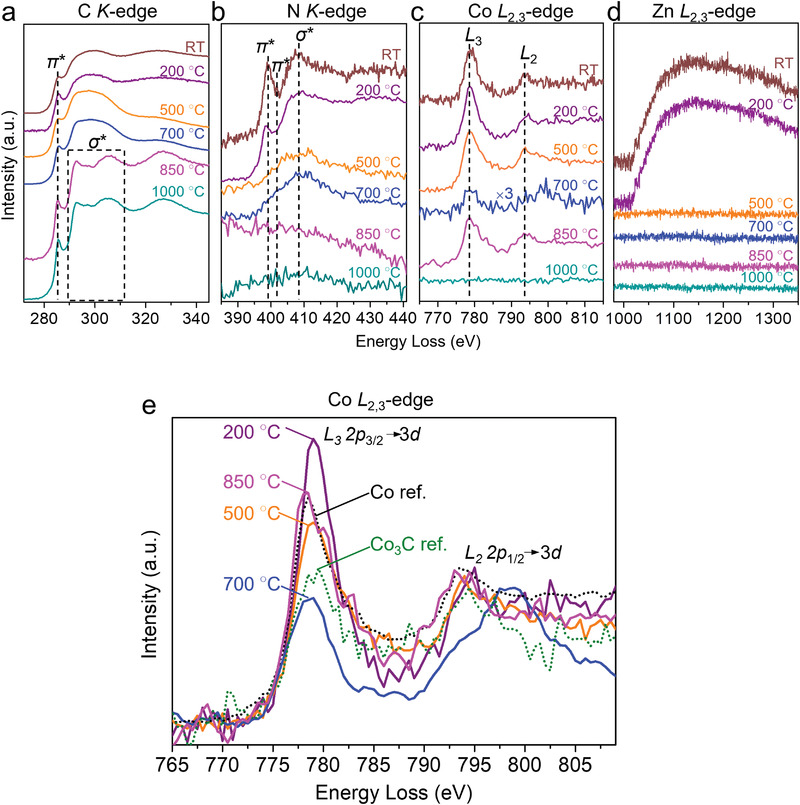
In‐situ STEM‐EELS characterization. a–d) In‐situ EELS spectra of C, N, Co, and Zn acquired at room temperature–1000 °C. All the spectra were calibrated by using the zero‐loss peak (0 eV). e) Normalized Co EELS of 200, 500, 700, and 850 °C‐pyrolyzed samples and references (metallic Co and Co_3_C^[^ ^54^ ^]^) measured at 400–600 °C. The spectra were calibrated with respect to the Co *L*_2_ energy loss peak after energy calibration using the zero‐loss peak (0 eV). The Co EELS obtained at 700 °C was smoothed from original spectrum as shown in (c).

We measured the signals originated from the *K* edges of C and N, and the *L* edges of Co and Zn. The Zn *L*‐edge signal was not detected at 500 °C, indicating the disappearance of Zn due to evaporation prior to this point (**Figure** **2**d). Graphitization of the ZIF precursor started at 850 °C. The electron energy loss peak at 284 eV shown in Figure 2a corresponds to the C *K*‐edge 1*s*−π* transition, while the multiple peaks in the range of 290−310 eV can be attributed to the 1*s*−σ* transition. These peaks are characteristic of *sp* ^2^‐hybridized carbon in a graphitic structure.^[^ ^78^ ^]^ In the N *K*‐edge region, when pyrolyzed from room temperature to 700 °C, the imidazole peaks of the ZIFs progressively converting to pyridinic and graphitic peaks,^[^ ^31^, ^78^, ^79^ ^]^ indicating a likely transformation of the Co‐N_4_ complex within the ZIFs to active Co‐N*_x_* sites embedded in carbon. However, upon further increase of temperature, the N‐based signals decreased significantly and became undetectable above 850 °C due to the putatively low content of N and the limited resolution of the instrument. This result is consistent with literature reports of N loss at high temperatures.^[^ ^80^ ^]^ However, the residual amount of N can still be detected by EDX characterization that showed the existence of N after 1000 °C ETEM experiment (Figure 1l and Figure S5, Supporting Information), thus proving the stability of C—N under the conditions used for pyrolysis. The in‐situ EELS of Co obtained from room temperature to 1000 °C is shown in Figure 2c. The EELS of the sample pyrolyzed at 1000 °C is essentially featureless in the Co *L*_2,3_ peak range due to the low Co content. Instead, STEM and EDX were used to verify the existence of atomically dispersed Co on the carbon support (Figure 1l,m and Figures S5 and S6, Supporting Information).

The EELS spectra of Co species obtained in the 200 to 850 °C temperature range were normalized and then compared with those of metallic Co and Co_3_C that serve as references. Specifically, the normalized EELS of Co species at 500 °C exhibited an *L*_3_/*L*_2_ ratio that is lower than that of the sample pyrolyzed at 200 °C but close to that of the metallic Co reference (Figure 2e); the reduction of Co(II) of Co/Zn‐ZIF to metallic Co at 500 °C can then be reasonably inferred with carbon or other carbon‐hydrogen species generated during the pyrolysis acting as the reductant.^[^ ^54^ ^]^ At 700 °C when small cobalt nanoparticles disappeared and digested within the support (Figure 1c), the atomically dispersed Co in carbon support showed a similar valence state (i.e., similar intensity ratio of *L*_3_/*L*_2_ energy loss peak) to Co_3_C but it is not the Co_3_C particle form. This result suggests that the atomically dispersed Co interacted with surrounding C. The Co—C interaction is not strong because the graphite CN*_x_* (anchor site) did not yet generate at 700 °C (see Figure 2a, C *K*‐edge EELS of 700 °C). Therefore, further increase of temperature to 850 °C caused re‐aggregation of Co atoms into nanoparticles that showed a similar valence state to metallic Co. The Co‐EELS evolution indicated that the atomically dispersed Co on support formed at 700 °C was thermally unstable and can further be aggregated at a higher temperature.

### 2.3 Formation of Single Co Atoms Anchored onto Porous N‐Doped Carbonaceous Support

To reveal the formation of single Co atoms on porous structural support, we followed the pyrolysis process at 850 °C and found that single Co atoms were produced and anchored onto a porous N‐doped carbonaceous support formed concomitantly.

**Figure 3:**
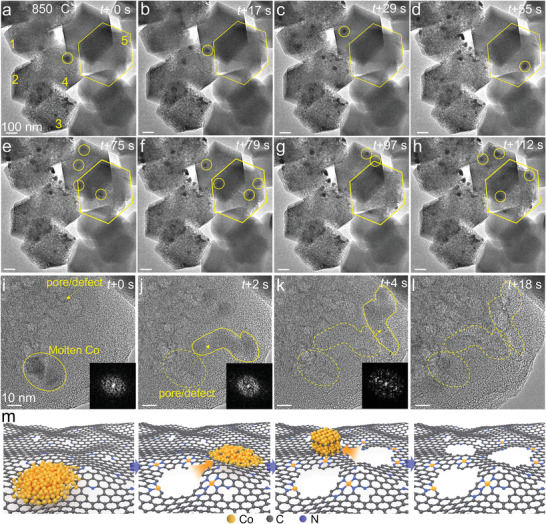
In‐situ ETEM showing the molten Co etching ZIF to produce porous structure CN*_x_* at 850 °C. a–h) Time‐sequenced ETEM images extracted from Video S2, Supporting Information, showing the molten Co etching ZIF. i–l) Close‐up view of molten Co etching ZIF extracted from Video S3, Supporting Information. Inset: FFT patterns derived from particle region were also shown. The trajectory of mobile Co droplet is marked by circle and arrow. m) Schematic of molten Co etching ZIF, formation of porous structure, and Co atom anchored by CN*_x_*.

The time‐sequenced ETEM images reveal the etching of the original imidazolate support by free‐moving Co nanoparticles at 850 °C (**Figure** **3**a–h, extracted from Video S2, Supporting Information). At 0 s, a number of porous structures (numbered 1–4) have already appeared, together with some not fully converted ZIFs (numbered 5, marked by frame) (Figure 3a). It is of particular note that Co nanoparticles migrated freely throughout the framework structure. As the process progressed (between 55 and 112 s), a porous structure onto which Co single atoms anchored appeared as a result of the etching of the framework structure (numbered 5) by the mobile Co nanoparticles (Figure 3d–h). Figure 3i–l extracted from Video S3, Supporting Information, shows a close‐up view of the etching process which is also schematically illustrated in Figure 3m. The mobile Co with irregular morphology, behaving like a liquid droplet, migrated randomly on the support. Fast Fourier transformation (FFT) of the mobile nanoparticles did not reveal any diffraction point; the molten state of the cobalt nanoparticles is thus further confirmed (Figure 3i–k). These nanosized Co nanoparticles melt at 850 °C, lower than the melting point of bulk form Co (1495 °C) due to the size‐dependent melting of small particles that the melting point of metal nanoparticles significantly decreases with decreasing particle size.^[^ ^48^ ^]^

To identify the composition of the molten nanoparticles, the temperature was lowered to 800 °C, and lattice fringes characteristic of metallic Co were observed (Figure S7, Supporting Information). A similar etching process (Figure S8, Supporting Information) to those shown in Figure 3i–l indicates that the etching of the framework structure by the mobile Co nanoparticles is indeed the general route to the final catalyst structure featuring Co single atoms anchored onto a porous N‐doped carbonaceous support. To exclude any possible effect due to e‐beam irradiation, we also performed ETEM at 850 °C with beam off and then captured the TEM images with the beam on. A similar observation of the porous structural support formed by way of mobile Co nanoparticles etching the framework structure was made (Figure S9, Supporting Information).

Our in‐situ ETEM and EELS results clearly show that the Co‐containing molten state at 850 °C with the etching of the framework occurred as the mobile nanoparticles moved within the structure. Formation of the porous structure is believed to be the consequence of moving Co nanoparticles etching carbon support and simultaneously carbon dissolving into the Co nanoparticles to form carbides as expressed in the following equation:

(1) $$\mathrm{Co}\left(l\right)+C\left(\mathrm{ZIF}\right)\to \mathrm{Co}C_{x}+C_{1-x}\left(\mathrm{defect}/\mathrm{porous}\;\mathrm{structure}\right)$$

The Brunner–Emmet–Teller (BET) analysis of the powder sample prepared with this melting‐assisted procedure shows a high surface area (729.5 m^2^ g^−1^) (Figure S10, Supporting Information), demonstrating the porous carbonaceous structure.

The above dynamic process resulting in Co SAC supported on the porous structure is consistent with the conclusion reached by previous experimental^[^ ^53^, ^54^ ^]^ and theoretical^[^ ^49^, ^81^, ^82^ ^]^ works that demonstrate the dissolution of carbon into metal (Fe, Co, Ni) nanoparticles at high temperatures. Moreover, computational studies predict that a metal droplet favors wetting on a carbon substrate when its carbon content (*χ*_C_) is low, while a higher *χ*_C_ favors a de‐wetting behavior.^[^ ^57^, ^83^ ^]^ As such, motion of the nanoparticle was mitigated with the increased amount of dissolved C and eventually stopped (Figure 3a–h) with the formation of Co_2_C/Co_3_C nanoparticles supported by the etched and porous carbonaceous structure (Figure S11a,c, Supporting Information). We did not observe the obvious sublimation of these de‐wetting nanoparticles at 850 °C even when heating for a long time (Figure S11b, Supporting Information). However, the de‐wetting particles were sublimated and completely disappeared with prolonged heating at a higher temperature (1000 °C) (Figure 1j, and Figure S12 and Video S1, Supporting Information).

**Figure 4:**
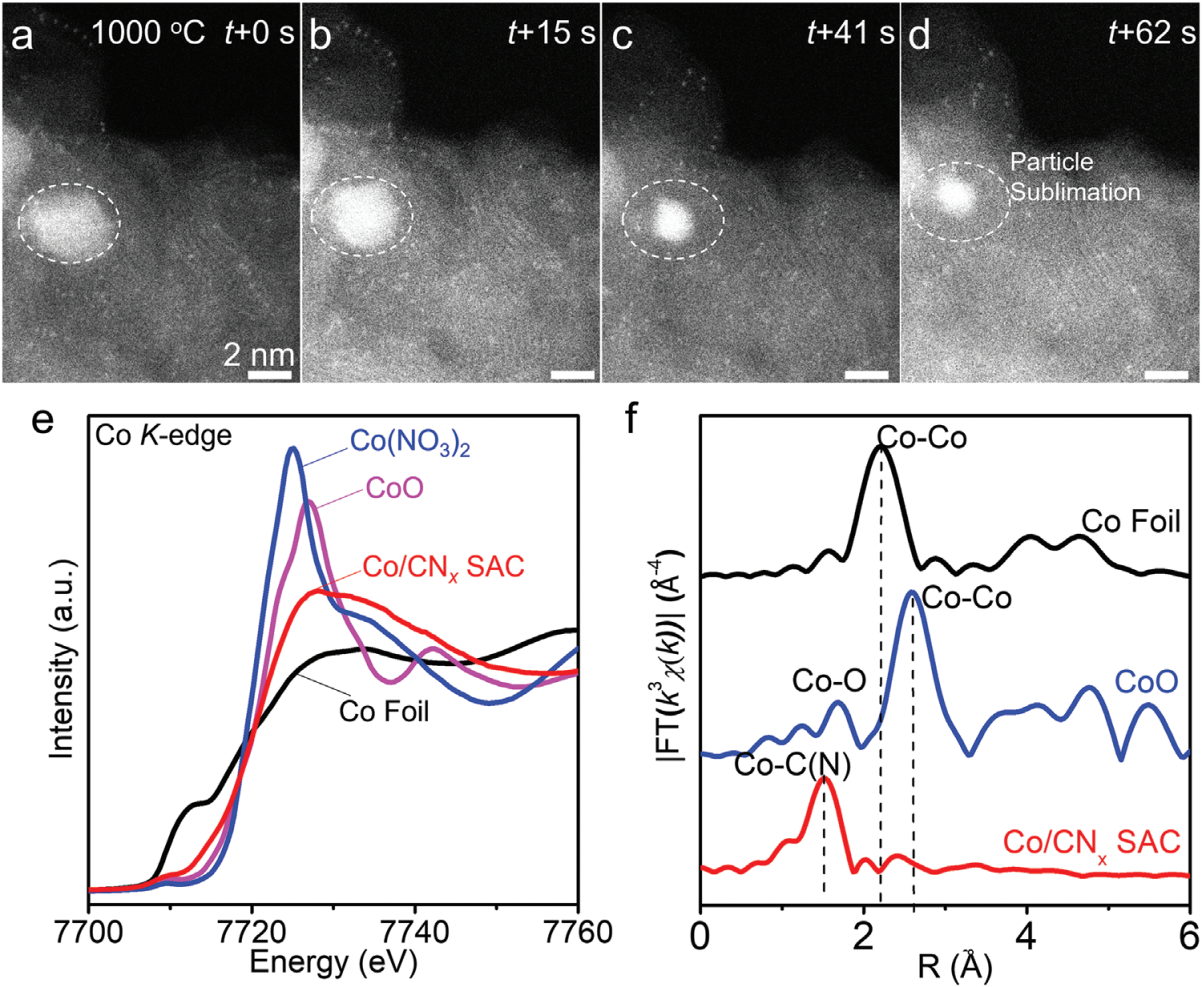
In‐situ HAADF‐STEM and XAS characterization of the stability of Co/CN*_x_* SAC. a–d) Time‐sequenced in‐situ HAADF‐STEM images at 1000 °C showing the sublimation of nanoparticles and the stability of single Co atoms on CN*_x_* support. The dashed circle is meant to help with observation of nanoparticle evaporation. e,f) The Co *K*‐edge XANES data (e) and Fourier transforms of EXAFS (f) of Co/CN*_x_* SAC. Co foil, CoO, and Co(NO_3_)_2_ are used as reference samples.

To further visualize the sublimation of nanoparticles and the stability of Co single atoms, in‐situ aberration‐corrected HAADF‐STEM at 1000 °C was adopted. The sample after 850 °C‐ETEM was further used for in‐situ HAADF‐STEM characterization, which comprised of Co single atoms and nanoparticles. It was found that de‐wetting nanoparticles underwent similar progressive sublimation (**Figure** **4**a–d, marked by dashed circles) to those shown in Figure 1f–j, whereas the Co single atoms were thermally stable on the porous support without any aggregation. This indicates that the single Co atoms were anchored by porous CN*_x_* support with strong metal–support interaction, which could be attributable to the electron transfer between Co and CN*_x_*. Additionally, we did not find the increase of Co single atoms during the evaporation of cobalt nanoparticles (Figure 4a–d), implying that the large nanoparticles possibly act as a spectator and do not involve in the formation of the SAC. This was further demonstrated by the control experiment where we annealed the physical mixture of Co nanoparticles and ZIF and found very few single Co atoms (Figure S13, Supporting Information). All these results indicates that the formation of Co/CN*_x_* SAC is not through a vapor phase deposition but the melting Co nanoparticles moving within the zeolitic architecture is essential to form single Co atoms anchored by C—N.

To probe the electronic interaction of the Co SAC by the synchrotron X‐ray absorption near‐edge spectroscopy (XANES), we also prepared the Co SAC bulk sample in tube furnace with the same method (see Experimental Section). We found that the bulk sample prepared in tube furnace at 800 °C exhibited the single‐atom state, as also demonstrated by aberration corrected HAADF‐STEM and XRD characterizations (Figure S14, Supporting Information). There exists a temperature difference between the Co SAC formed in ETEM (1000 °C) and tube furnace (800 °C), because ETEM chip and tube furnace have different heating modes.^[^ ^56^ ^]^ It is known that the sample is only heated on the micrometer‐scaled heating region of the ETEM chip and the surrounding (i.e., TEM chamber) is at the low temperature (similar to the cold‐wall furnace), so the evaporation of large nanoparticles in ETEM requires a higher temperature of 1000 °C. However, the surrounding is sufficiently heated in the hot‐wall tube furnace, so the evaporation of large nanoparticles is easier and occurs at a lower temperature in tube furnace (800 °C) than in ETEM (1000 °C). We found that the energy absorption edge and the height of white line are between those of Co(NO_3_)_2_, CoO, and Co foil (Figure 4e), indicating that the valence state of Co on the carbon support lies between Co(0) and Co(II). The Fourier transform (FT) *k* ^3^‐weighted extended X‐ray absorption fine structure (EXAFS) curve shows a main peak at 1.4 Å, corresponding to the first shell of Co—C(N) scattering,^[^ ^30^ ^]^ which is different from those of the CoO and Co nanoparticle references (Figure 4f). The XAS results demonstrate that Co atoms are individually anchored onto the support with strong electronic interactions between Co and the graphitic support. Such interactions are probably significant to the formation of the single atoms because a replacement for a non‐carbonaceous support (MgO, Al_2_O_3_, and SiO_2_) led to Co‐containing particles instead of Co‐single atoms as revealed by HAADF‐STEM images of the resulting catalyst (Figure S15, Supporting Information).

Additionally, we also correlated the number of single Co atoms to the actual loading of the Co in powder sample. The actual loading of Co (1.6 wt%, Table S1, Supporting Information) and specific surface area (792.5 m^2^ g^−1^, Figure S10, Supporting Information) in powder sample were measured by inductively coupled plasma mass spectrometry (ICP‐MS), EDX, and BET, respectively. Therefore, the density of single Co atom^[^ ^84^ ^]^ was estimated to be 0.21 atom/nm^2^ (see details in Supporting Information), which was in excellent agreement with measurements (0.2 atom/nm^2^) from HAADF‐STEM images (52 atoms per 256 nm^2^) (Figure S14, Supporting Information).

### 2.4 Selective Oxidation Catalysis

**Figure 5:**
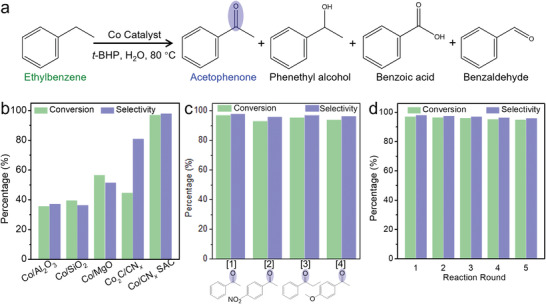
Catalytic performance of Co catalysts for selective oxidation of ethylbenzene to acetophenone. a) Selective oxidation of ethylbenzene by *tert*‐butyl hydroperoxide (*t*‐BHP). b) Catalytic performance of Co nanoparticles supported on Al_2_O_3_, SiO_2_, MgO, Co_2_C/CN*_x_*, and Co/CN*_x_* SAC. c) Selective oxidation of a series of aromatic alkane derivatives by Co/CN*_x_* SAC. Reaction conditions: [1] 1.0 mL *t*‐BHP, 10 h; [2–4] 2.0 mL *t*‐BHP, 11 h. d) Reusability of Co/CN*_x_* SAC.

The activation of the C—H bond in hydrocarbons by transition metal catalysts remains one of the most challenging reactions.^[^ ^85^ ^]^ The liquid phase oxidative conversion of ethylbenzene is attracting substantial attention to produce higher‐valued acetophenone which is an important intermediate for chemical industries. Oxidation of ethylbenzene was performed as a model reaction (**Figure** **5**a) to evaluate the obtained SAC in hopes of correlating the uniquely structured catalyst with its properties.

We found that the catalyst powder pyrolyzed from Co/Zn‐ZIF in tube furnace at 800 °C showed the similar single Co atom state to that in 1000°C‐ETEM as evidenced by HAADF‐STEM and EDX (Figure S14a–g, Supporting Information). The content of Co (1.6 wt%) in final Co/CN*_x_* SACs (Table S1, Supporting Information) is lower than that in Co/Zn‐ZIF precursor (Co 6.2 wt%), further demonstrating the evaporation of Co nanoparticles during pyrolysis. The Co/CN*_x_* SAC‐800°C powder catalyst exhibits a remarkably higher ethylbenzene conversion (97%) and selective production of acetophenone (98%) than those non‐single‐atom powder catalyst pyrolyzed at lower temperatures (500–700 °C) (Figure S16, Supporting Information). When further increasing the catalyst pyrolysis temperature to 900 °C in furnace, similar catalytic performance was achieved (Figure S16, Supporting Information). These results indicate the stability of Co/CN*_x_* SAC at high temperature. Figure 5b compares the catalytic performance of various Co catalysts supported on oxides and CN*_x_* prepared at 800 °C. No ethylbenzene conversion was observed in the control experiment without any catalyst. The Co/CN*_x_* SAC‐800°C powder catalyst exhibited remarkably higher ethylbenzene conversion and selective production of acetophenone than when Co nanoparticles formed on oxides (Al_2_O_3_ SiO_2_, and MgO) (Figure 5b and Figure S15, Supporting Information) were used in otherwise identical reactions. To exclude the catalytic contribution of Co*_x_* C nanoparticles, we prepared the Co_2_C nanoparticles (Figure S17, Supporting Information) and supported on CN*_x_* (see Experimental Section), which was used as a control catalyst to evaluate the reaction under the same conditions. The controlled Co_2_C/CN*_x_* catalysts showed lower ethylbenzene conversion and acetophenone selectivity than Co/CN*_x_* SAC (Figure 5b), further demonstrating the advantage of Co/CN*_x_* SAC. The present Co/CN*_x_* SAC catalyst was also proved effective in the selective oxidation of other aromatic alkanes, with conversion ≥93.0% and selective production of ketone ≥96.0% (Figure 5c). Essentially undiminished results were achieved during 5 reaction cycles using the catalyst recovered after each run of evaluation (Figure 5d). These results indicate clearly the stability and unique catalytic properties of the present SACs.

## 3 Conclusion

The in‐situ TEM, STEM, and EELS results provide direct evidence of the microstructural and chemical evolution of Co/Zn‐ZIF during pyrolysis, producing nanoclusters and nanoparticles of Co as pyrolysis progressed. Surprisingly, upon further increase of temperature, the nanoparticles underwent a melting process with the formation of C‐containing clusters due to the dissolution of C of the ZIF into the nanoparticles. Specifically, four distinct steps are involved in this process: i) cobalt species was reduced and aggregated to metallic Co clusters by reductant pyrolyzed from ZIF at 500 °C; ii) the Co species was well‐dispersed on support at 700∼800 °C; iii) the atomically dispersed Co species re‐aggregated to nanoparticles and molten Co droplet moved and etched ZIF derivative to produce porous structure CN*_x_* with anchoring Co atoms at 850 °C; and iv) the rest cobalt carbide nanoparticles were sublimated at 1000 °C. This process is accompanied with the change of wettability of the Co‐containing species, resulting in a random motion of the nanoparticles on the carbonaceous support that stopped when a sufficient amount of C was dissolved. The formation of Co SAC was to demonstrate that through melting Co nanoparticles move within the zeolitic architecture with single Co atoms anchored onto a porous CN*_x_*, instead of a vapor phase deposition during the sublimation of nanoparticles, which has never been observed before. The unique catalytic properties of the Co‐SAC have been exemplified in the oxidation of ethylbenzene and its derivatives, resulting in the selective formation of the corresponding ketones.

The mechanistic insights gained by the in‐situ experiments for the evolution of the SAC are expected to be useful for the preparation of SACs of other transition metal elements by regulating the carbon solubility in the clusters or nanoparticles of metal at judiciously chosen pyrolysis temperatures. On the other hand, aberration corrected ETEM combined EELS may be widely applicable for studying the dynamics of heterogenous catalysis when multi‐scaled metal catalysts are involved.

## 4 Experimental Section

### Preparation of Co/Zn‐ZIF

A solution prepared by dissolving Zn(NO_3_)_2_·6H_2_O (0.558 g, 1.8 mmol) and Co(NO_3_)_2_·6H_2_O (0.546 g, 1.8 mmol) in 15 mL of methanol was added to a 15‐mL methanolic solution of 2‐methylimidazole (0.616 g, 7.5 mmol). This solution mixture was stirred at room temperature for 12 h. The resulting purple‐colored precipitate was collected by centrifugation, washed with methanol, and dried at 60 °C for 12 h in vacuo.

### ETEM Experiments

The in‐situ TEM experiments were conducted by using an aberration‐corrected Titan G2 80–300 ETEM. The ETEM chip with SiO_2_/SiN*_x_* membrane was mounted onto a Micro Electron‐Mechanical System based micro heater (ThermoFisher Scientific, NanoEx‐i/v). A sample of Co/Zn‐ZIF dispersed in ethanol was dropped onto the ETEM chip and then heated to a chosen temperature under high vacuum. Corresponding TEM images and videos were subsequently taken (electron dose‐rate: 0.8–4.7 e^−^·Å^−2^·s^−1^).

### In‐Situ EELS Experiments

In‐situ STEM‐EELS data were collected using the same aberration‐corrected ETEM. It was equipped with a Gatan image filter (Quantum 936) with an energy dispersion of 0.25 eV operated at an acceleration voltage of 300 kV. The zero‐loss EELS was acquired immediately after the obtainment of the core‐loss EELS on individual nanoparticles. The EELS data so obtained were further analyzed by Digital Micrograph. The position of the core‐loss EELS was corrected with the corresponding zero‐loss peak followed by subtracting the extrapolated background from the edge of interest. A Fourier‐ratio deconvolution was further performed to remove the effect of plural scattering.

### In‐Situ HADDF‐STEM Experiments

The in‐situ HADDF‐STEM experiments were performed on an aberration‐corrected STEM (FEI Titan Cubed Themis G2 300) equipped with Cs double corrector DCOR and a high‐brightness field emission gun (X‐FEG), which was operated at an acceleration voltage of 300 kV. The sample obtained by pyrolysis of Co/Zn‐ZIF at 850 °C and used for the above ETEM experiment was heated with the beam off to 1000 °C in Cs‐STEM at a rate of 1 °C s^−1^ under high vacuum. The STEM images were then taken with the beam on.

### Preparation of Co/CN*_x_* Powder Sample

A quartz boat containing a sample of Co/Zn‐ZIF was placed in a tube furnace, and the setup was heated under N_2_ to 500–900 °C at a rate of 2 °C min^−1^. The pyrolysis process was maintained at this temperature for 1 h, and the content was cooled naturally to room temperature to afford the Co/CN*_x_* SAC used for subsequent catalytic oxidation of ethylbenzene.

### Preparation of Co/Al_2_O_3_, Co/SiO_2_, and Co/MgO

Typically, the Co/Al_2_O_3_, Co/SiO_2_ and Co/MgO catalyst was prepared by an incipient wetness impregnation method. The aqueous solutions of Co(NO_3_)_2_·6H_2_O (0.273 g) and commercial Al_2_O_3_, SiO_2_ and MgO (36.4 g) were stirred at room temperature for 1 h, respectively. Subsequently, the mixture was dried an oven at 80 °C overnight. The obtained catalyst precursor was calcined in a quartz tube furnace under N_2_ to 800 °C at a rate of 2 °C min^−1^ for 1 h and cooled naturally to room temperature.

### Preparation of Co_2_C/CN*_x_*

The synthesis of Co_2_C nanoparticles was accomplished using the polyol process. In a typical experiment, 0.30 g cobalt chloride anhydrous (CoCl_2_), 0.52 g KOH, and 50 mL tetraethylene glycol were placed in were placed in a three‐neck flask. Then the mixture was heated at 290 °C for 30 min under reflux, with magnetic stirring and N_2_ gas flow to remove most of the ambient atmosphere. The solution was cooled to room temperature and the particles were magnetically separated and were then washed numerous times with ethanol and dried at room temperature in a vacuum oven.

### XAS and BET Measurements

The XAS measurements were carried out at Beijing Synchrotron Radiation Facility (BSRF 1W1B beamline). This beamline adopted fixed‐exit double crystal Si (111) monochromator to achieve an X‐ray energy range of 5–23 keV. The X‐ray beam size on the sample is ca. 0.5 × 0.25 mm with flux higher than 10^10^ photon/s. The Co *K*‐edge XANES data were recorded in a fluorescence mode using a Lytle detector. Co foil was used as reference to calibrate the absorption energy. The samples were grinded and uniformly daubed on an adhesive tape. The data of XANES and extended X‐ray absorption fine structure (EXAFS) were analyzed by Athena and Artemis software. The nitrogen adsorption measurement was characterized by the ASAP 2020 System and the surface area of the powder samples was calculated by the BET method.

### Selective Oxidation of Ethylbenzene

In a typical experiment, with magnetic stirring at 1500 rpm, a 25‐mL pressure‐resistant glass tube charged with Co‐SAC (10 mg), ethylbenzene (61 µL, 0.5 mmol), H_2_O (1 mL), and *t*‐BHP (70% solution in water, 1 mL) was maintained at 80 °C for 10 h. After cooling to room temperature, anisole (77 µL) was injected into the tube as an internal standard, and the resulting mixture was extracted thoroughly with ethyl acetate (10 mL). The extracted organics were subsequently analyzed by using a Fuli gas chromatograph equipped with an FID detector with N_2_ as carrier gas.

## Conflict of Interest

The authors declare no conflict of interest.

## Author Contributions

F.Y. wrote the manuscript and Z.Z. revised the manuscript. L.Z. and L.Z. prepared the TEM sample, collected, analyzed ETEM/EELS data, and performed the catalysis. X.Z. and Y.L. prepared the ZIF sample and performed the ex‐situ characterizations. All authors contributed to data analysis, interpreted the data, and approved the final manuscript. L.Z., Y.L., and L.Z. contributed equally.

## Supporting information

Supporting Information

Supplemental Video 1

Supplemental Video 2

Supplemental Video 3

## Data Availability

The data that support the findings of this study are available from the corresponding author upon reasonable request.
