# Supplementary material for: Direct Visualization of the Evolution of a Single‐Atomic Cobalt Catalyst from Melting Nanoparticles with Carbon Dissolution
Source: Adv Sci (Weinh). 2022 May 4;9(20):2200592. doi: 10.1002/advs.202200592 (PMC9284138; doi:10.1002/advs.202200592)
Supplement: Supplementary file 1 — Supporting Information [file ADVS-9-2200592-s004.pdf]

ADVANCED SCIENCE
Open Access

Supporting Information

for *Adv. Sci.*, DOI 10.1002/advs.202200592

Direct Visualization of the Evolution of a Single-Atomic Cobalt Catalyst from Melting Nanoparticles with Carbon Dissolution

Luyao Zhang, Yanyan Li, Lei Zhang, Kun Wang, Yingbo Li, Lei Wang, Xinyu Zhang, Feng Yang* and Zhiping Zheng**

Supporting Information

Direct Visualization of the Evolution of a Single-Atomic Cobalt Catalyst from Melting Nanoparticles with Carbon Dissolution

Luyao Zhang,† Yanyan Li,† Lei Zhang,† Kun Wang, Yingbo Li, Lei Wang, Xinyu Zhang*, Feng Yang*, Zhiping Zheng*

This PDF file includes:

Figures S1–S17

Table S1

Other supplementary material:

Video file:

Video S1. mp4

Video S2. mp4

Video S3. mp4

Results

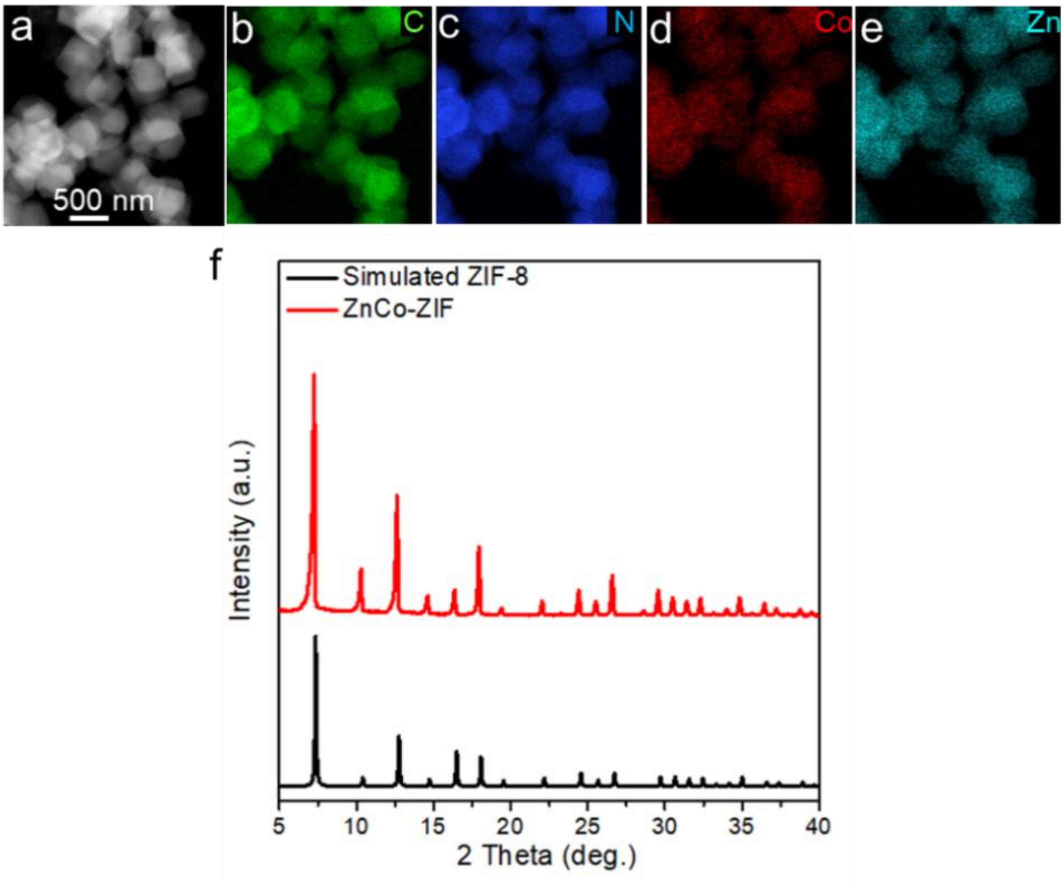

Figure S1. **a**–**e**, STEM-EDX mapping of Co/Zn-ZIF. **f**, The experimental XRD pattern and the corresponding simulation XRD pattern of Co/Zn-ZIF.

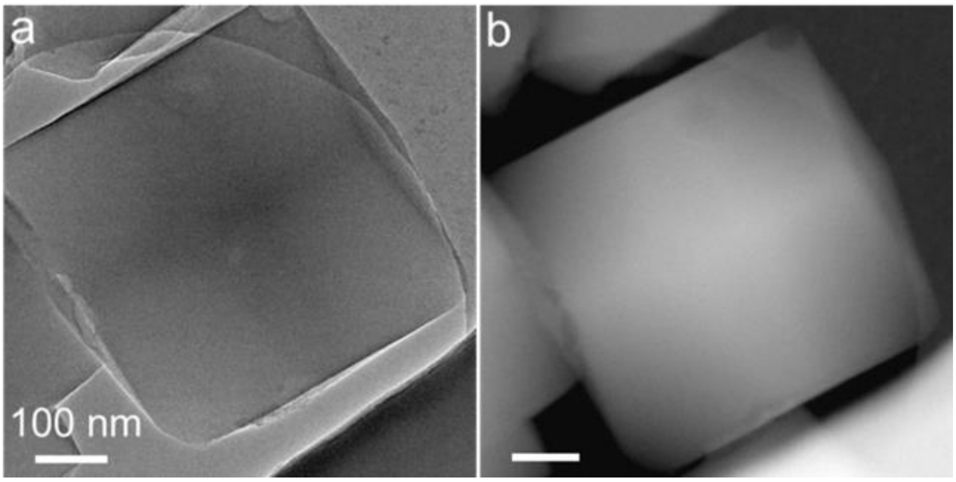

Figure S2. **a**, TEM and **b**, HAADF-STEM images of pristine ZIF at room temperature.

To reveal the evolution of Co species, we carefully analyzed the evolution of Co/Zn-ZIF pyrolyzed at 500–800 °C. Figure S3 shows the temperature-sequenced *in-situ* HAADF-STEM images from 500 °C to 800 °C and close-up view. The Co clusters formed at 500 °C exhibit similar size (2~5 nm) with those observed from *in-situ* TEM (Figure 1b, inset). We do not find single Co atoms at 500–800 °C in Figure S3e, f, which is also consistent with the *in-situ* TEM observation (Figure 1c, d). This implies that the clusters digested within the support at 700 °C.

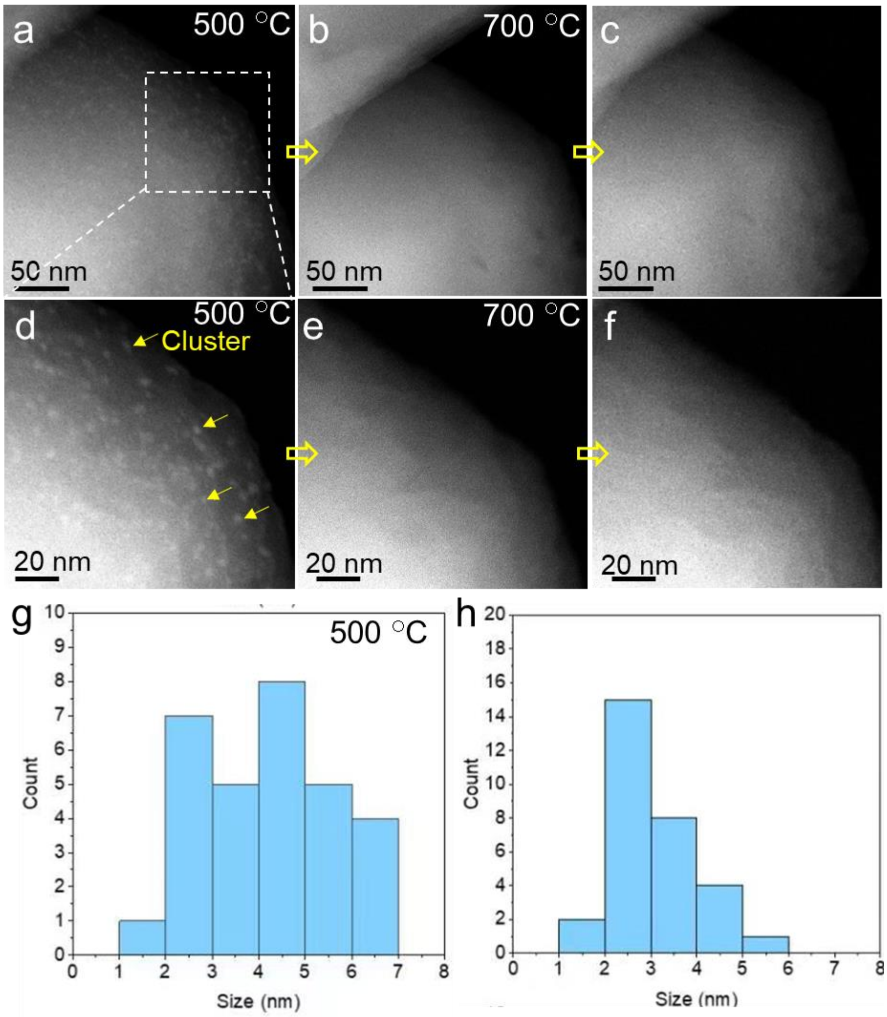

Figure S3. Temperature-sequenced *in-situ* HAADF-STEM images from 500 °C to 800 °C (**a**–**c**) and close-up view (**d**–**f**). **g**, **h**, Size distribution of clusters observed from HAADF-STEM (**g**) and TEM (**h**) at 500 °C. 30 and 30 numbers of randomly chosen clusters were used for statistical analyze, respectively. The size and counts of particle

number were analyzed by Digital Micrograph software.

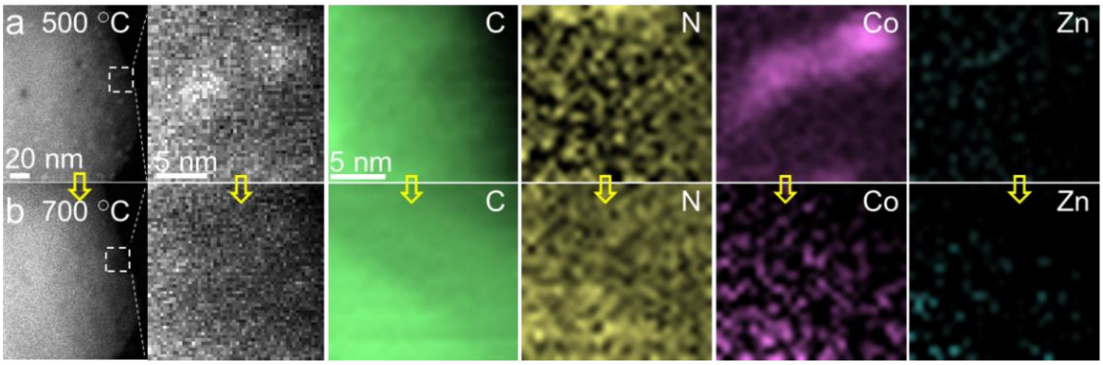

Figure S4. **a**, **b**, *In-situ* EELS spectra image, close-up view, and EELS elemental mapping of C, N, Co, and Zn at 500 °C (**a**) and 700 °C (**b**).

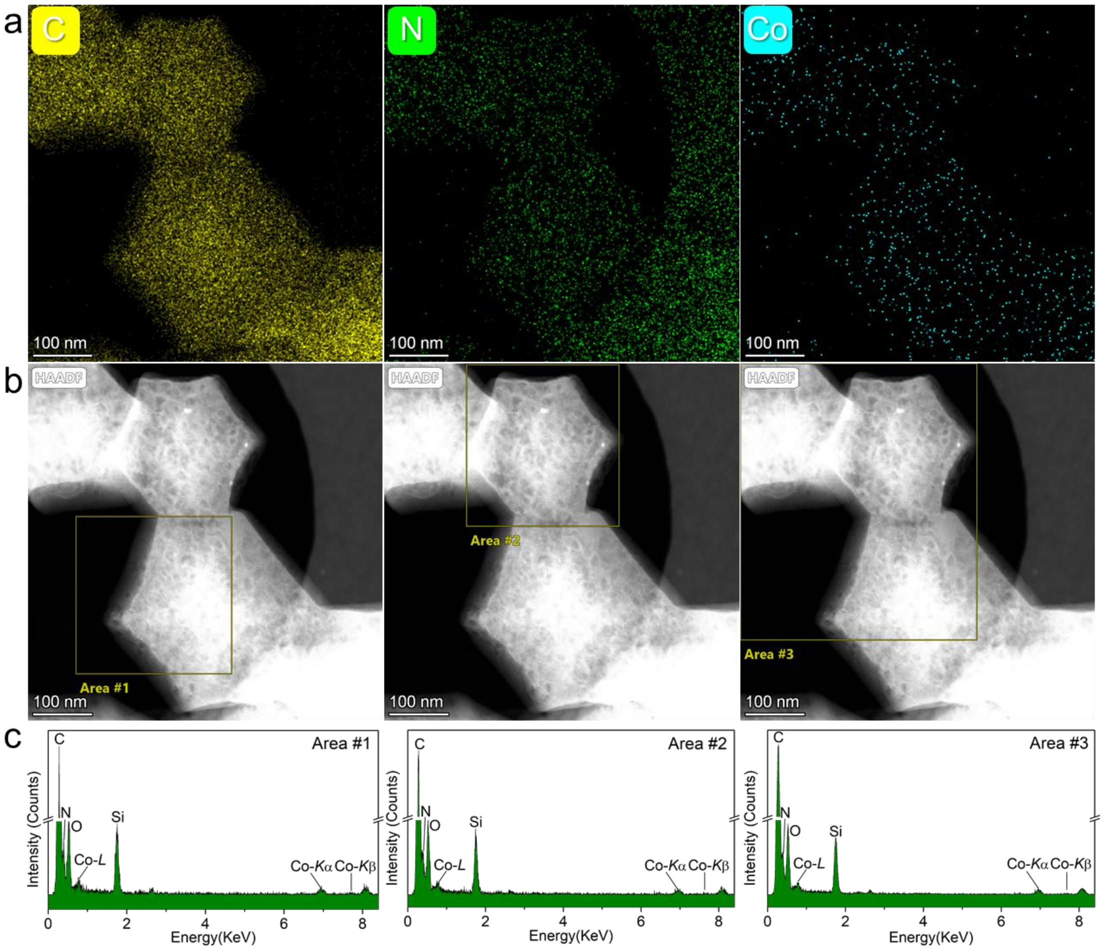

Figure S5. EDX characterization of sample after 1000 °C-ETEM experiment. **a**, EDX elemental mapping. **b**, HAADF-STEM images. **c**, EDX spectra acquired from three different regions marked in (**b**).

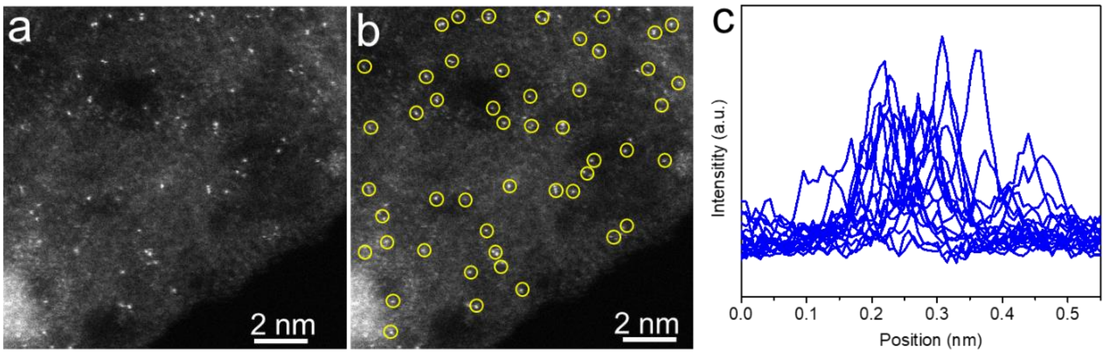

Figure S6. **a**, **b**, HAADF-STEM images without (**a**) and with (**b**) circles to identify the Co SAC after 1000 °C-ETEM experiment. **c**, Intensity profile from HAADF-STEM images showing the uniform intensity of Co SACs (**a**).

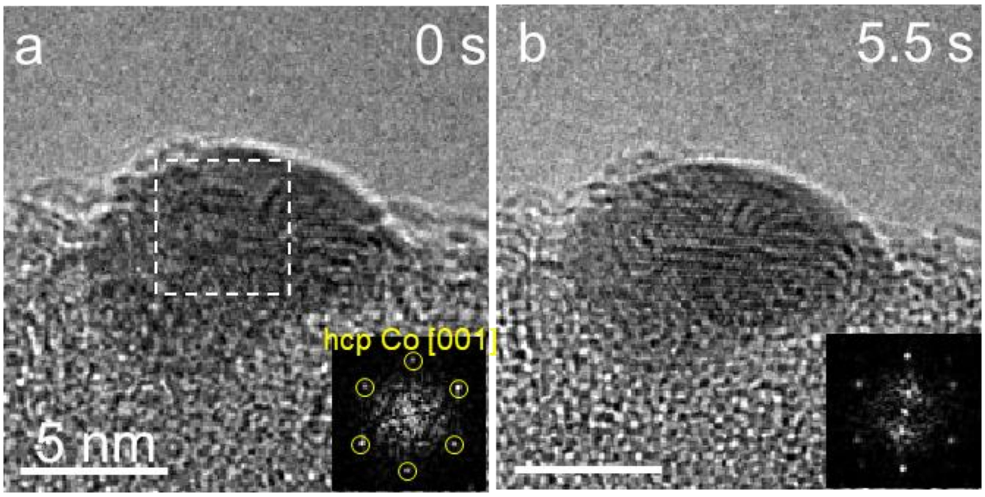

Figure S7. **a**, **b**, ETEM images acquired at 800 °C showing the metallic Co crystalline. Inset: FFT pattern of the particle.

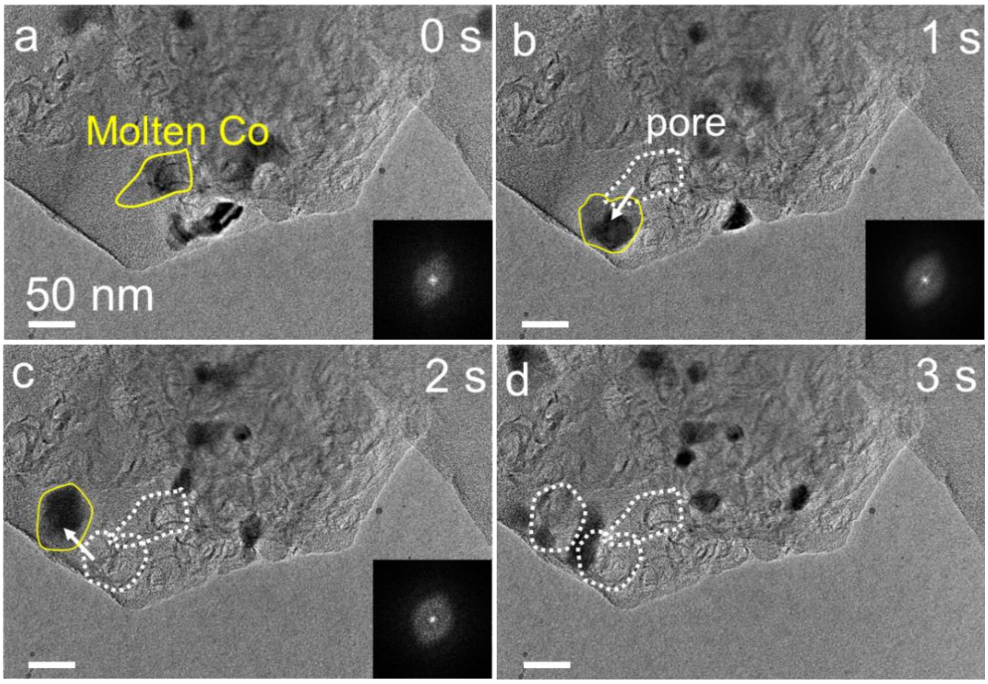

Figure S8. Time resolved *in-situ* TEM images of molten Co nanoparticles etching ZIF recorded at 850 °C. Inset: corresponding FFT pattern of the molten Co marked in yellow line.

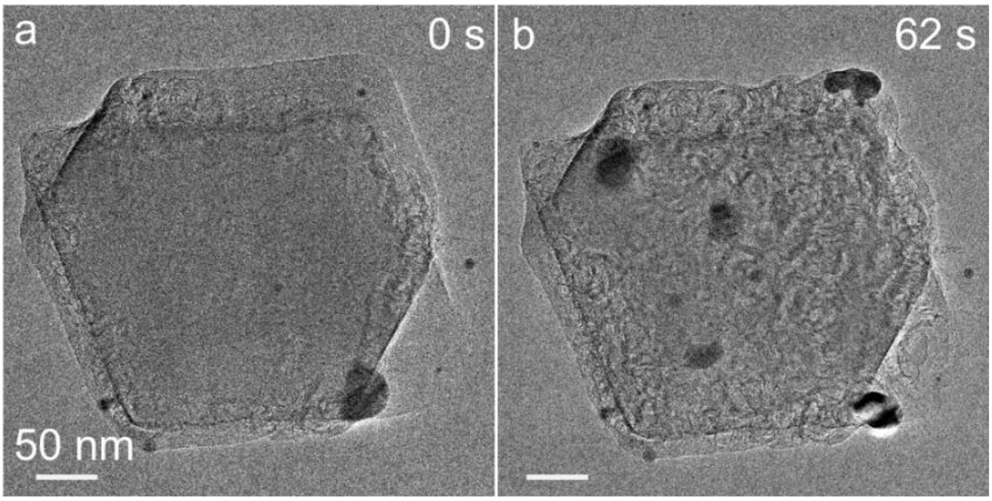

Figure S9. **a**, **b**, Time-sequenced *in-situ* TEM images of molten Co nanoparticles substrate recorded at 850 °C with electron-beam off for 1 min.

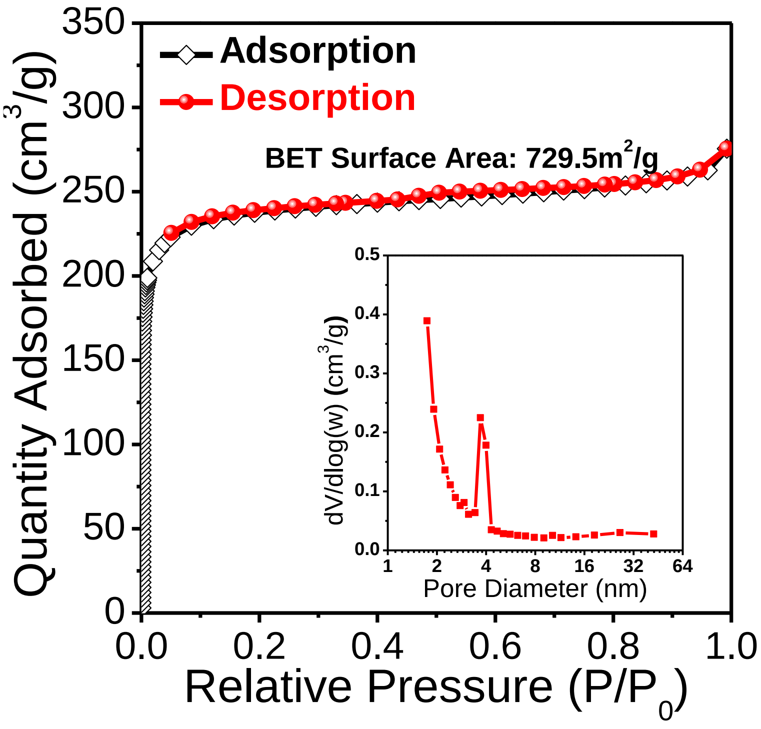

Figure S10. BET analysis of Co on porous carbonaceous support.

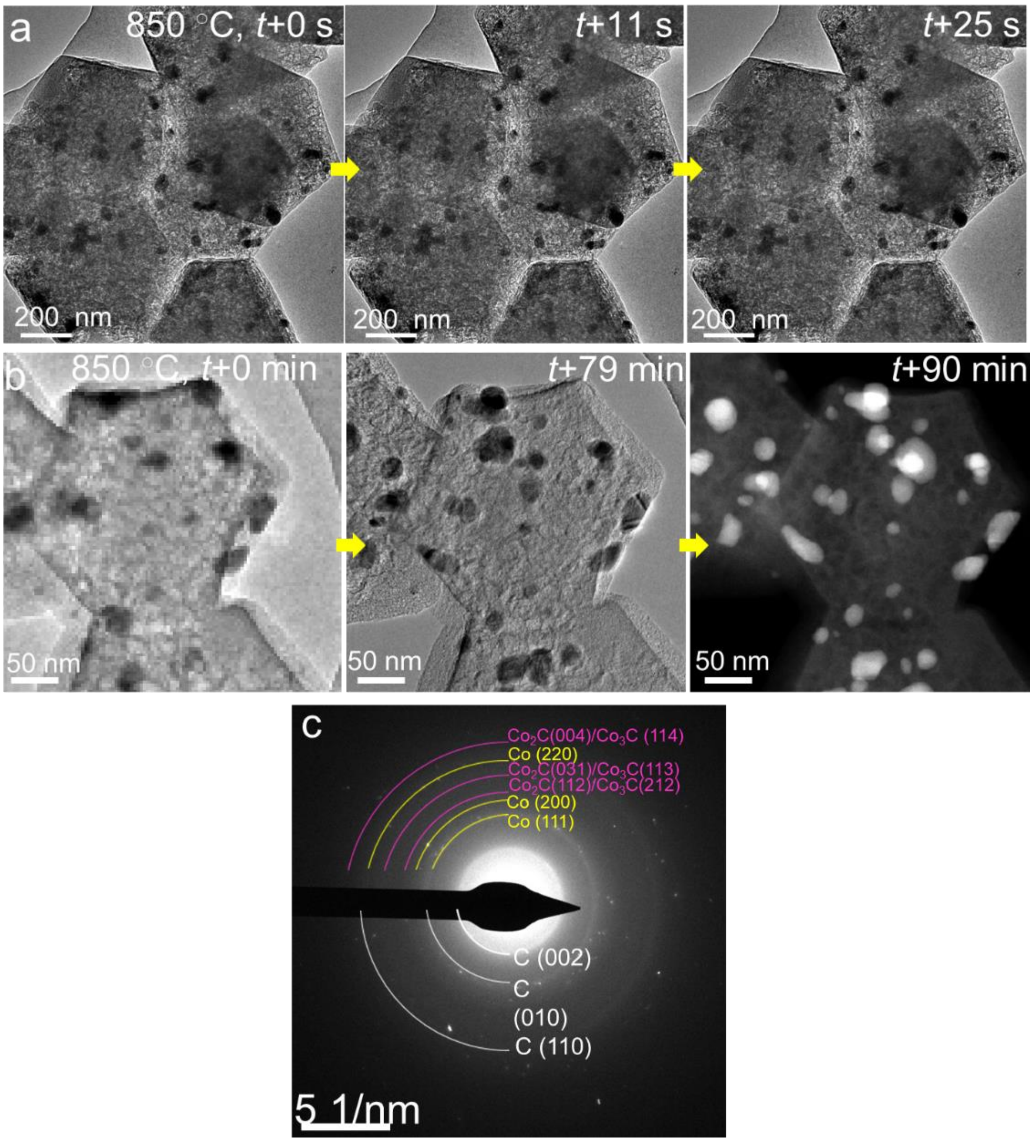

Figure S11. **a**, **b**, *In-situ* S/TEM images of nanoparticles on substrate at 850 °C for a short (**a**) and long time (**b**), showing that nanoparticles maintain stable after etching ZIF and do not sublimate at 850 °C. **c**, SAED pattern showing the mixture of Co and cobalt carbides.

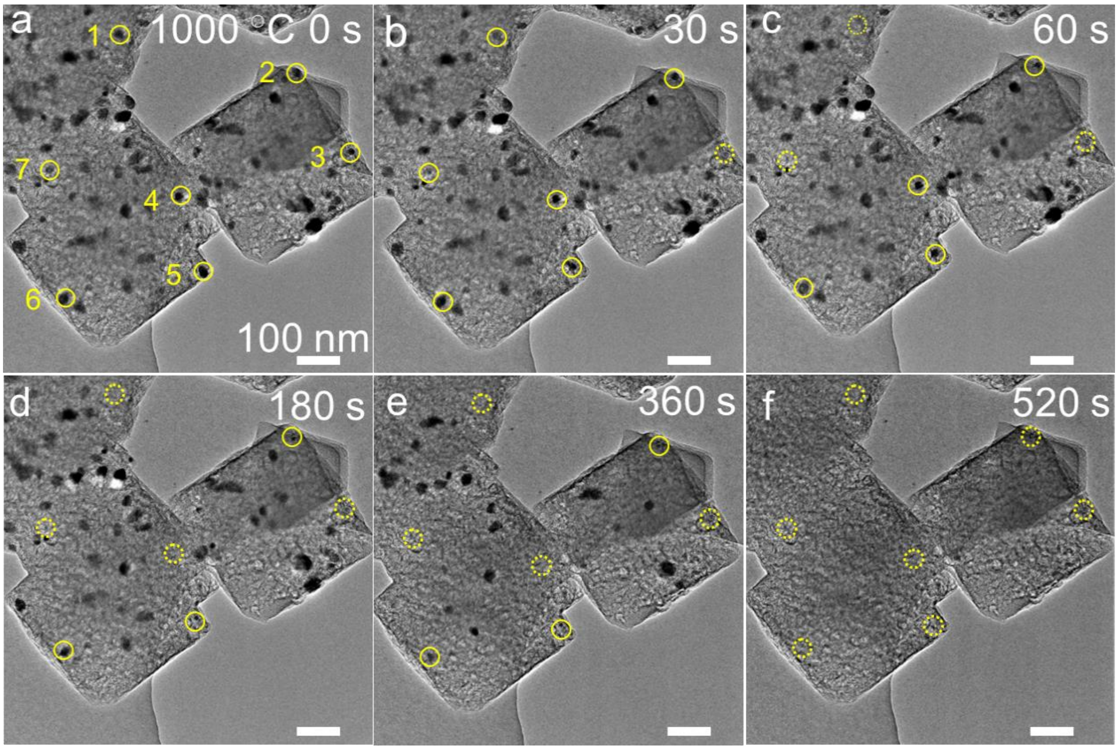

Figure S12. **a**–**f**, Time-sequenced ETEM images showing the sublimation of cobalt nanoparticles from CN_x recorded at 1000 °C. The circles are meant to help with comparison.

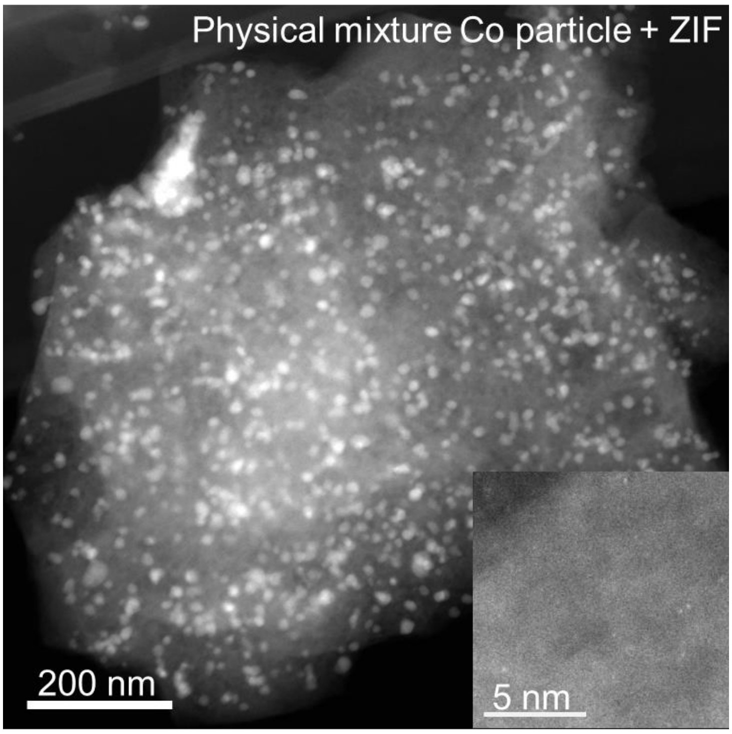

Figure S13. HAADF-STEM images of Co sample by annealing the physical mixture of Co nanoparticles and ZIF. Inset image shows very few single Co atom.

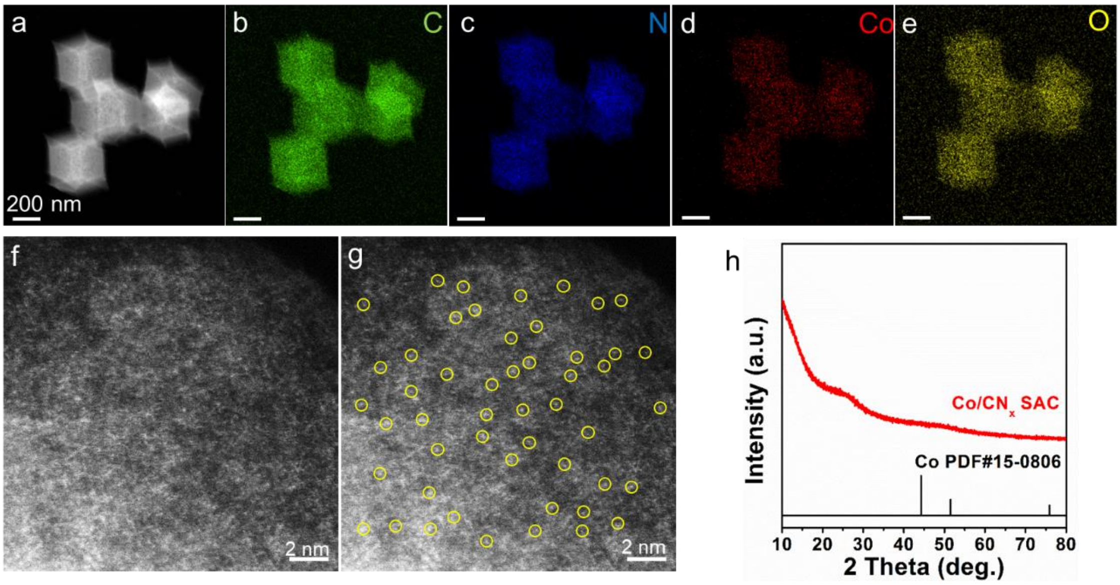

Figure S14. **a-e**, STEM-EDX characterization of SAC Co/CN$_x$ prepared in tube furnace. **f**, **g**, HAADF-STEM image SAC Co/CN$_x$ powder sample: without (**f**) and with circles (**g**) to help with observation of SAC. **h**, XRD pattern of Co SAC powder sample.

For the Co SAC powder sample prepared in tube furnace, there is no obvious diffraction peak by XRD, indicating the single Co atom state. The actual Co loading of the powder Co SAC sample was measured to be 1.5 wt.% by ICP analysis and the specific surface area was measured to be 729.5 m^2/g by BET. Therefore, the density of single Co atoms in powder sample was estimated to be 0.21 atom/nm^2 by the equation:

$$\text{Density of single Co atom} = \frac{\text{Co atom number}}{\text{Surface area}}$$

$$= \frac{\dfrac{1\ \text{g} \times 1.5\ \text{wt.\%}}{58.9\ \text{g/mol}} \times 6.02 \times 10^{23}}{1\ \text{g} \times 729.5 \times 10^{18}\ \text{nm}^2/\text{g}}$$

$$= 0.21\ \text{atom/nm}^2$$

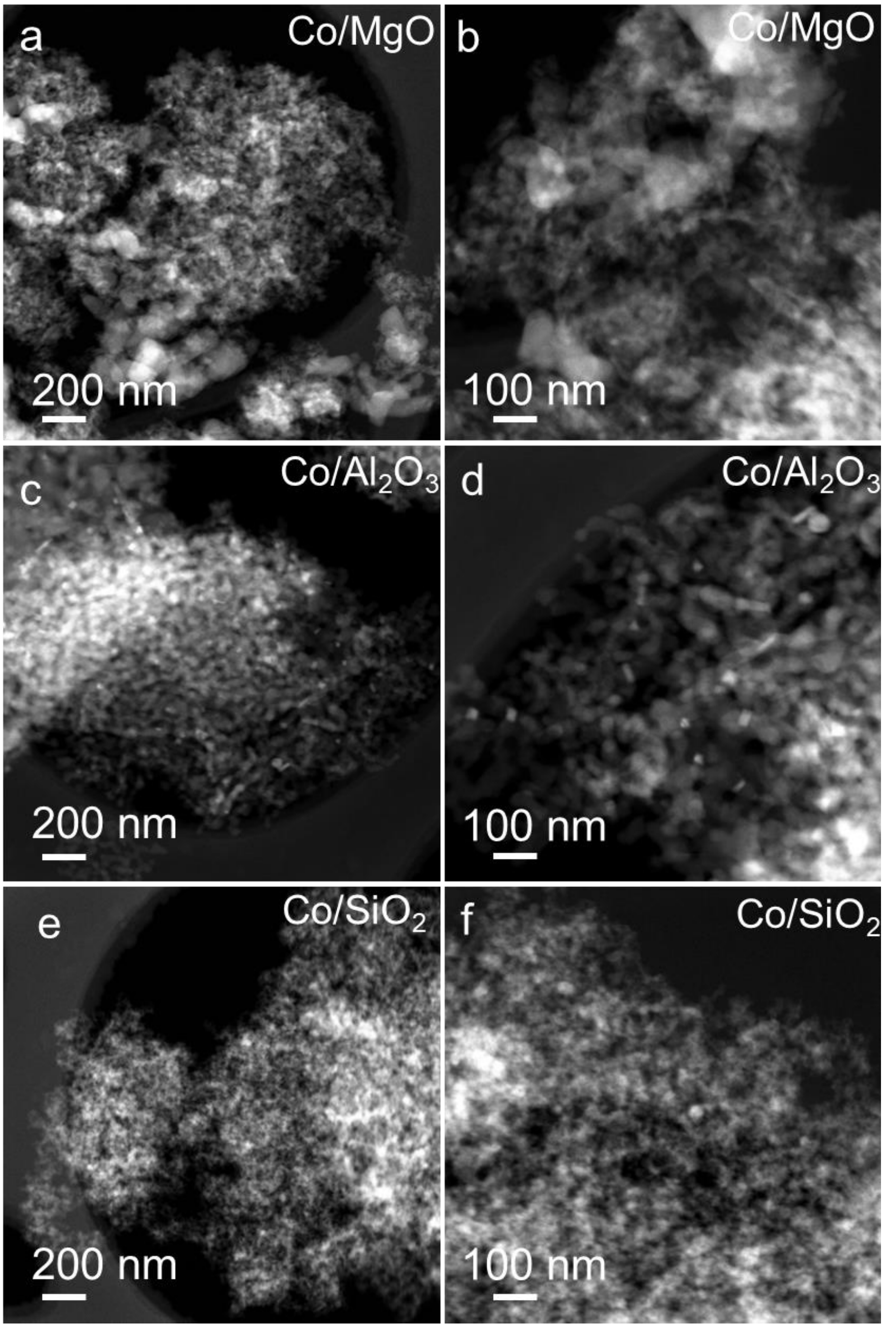

Figure S15. HAADF-STEM images of Co/MgO (**a**, **b**), Co/Al_2O_3 (**c**, **d**) and Co/SiO_2 (**e**, **f**) catalysts.

Table S1. Elemental analysis of Co-SAC from EDX, showing a Co loading of 1.6 wt.%.

Element	C	N	Co	O
Mass Fraction %	73.2	8.6	1.6	9.0

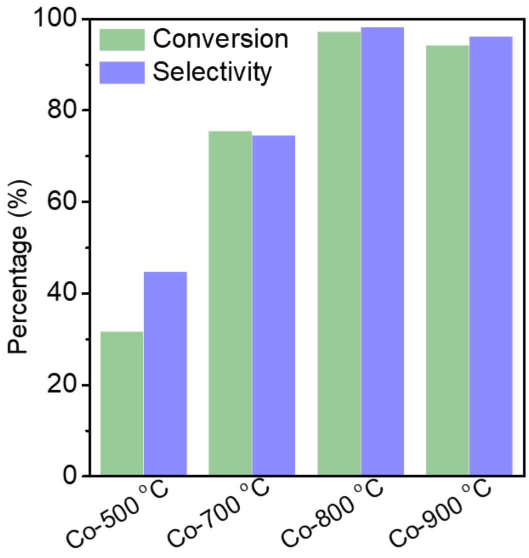

Figure S16. Catalytic performance of Co/CN$_x$ catalyst powder pyrolyzed from CoZn-ZIF in furnace at different temperatures (500–900 °C).

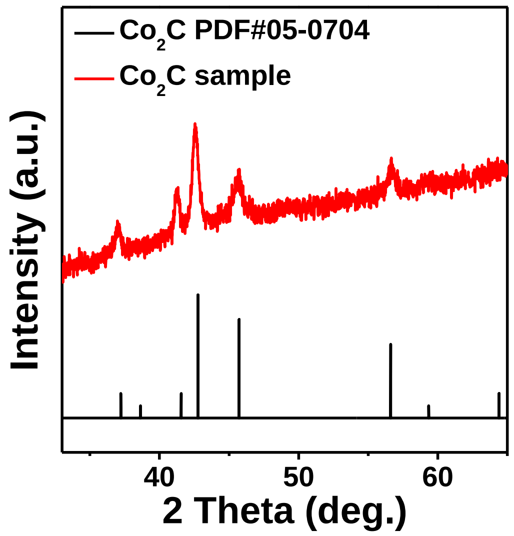

Figure S17. XRD patterns of as-prepared Co_2C nanoparticles.

Video captions

Video S1. *In-situ* TEM movie showing the gradual sublimation of cobalt nanoparticles at 1000 °C in Figure 1f–j. The movie is displayed at 30× speed.

Video S2. *In-situ* TEM movie showing the etching process of ZIF by molten Co nanoparticles at 850 °C in Figure 3a–h. The movie is displayed at 7× speed.

Video S3. *In-situ* TEM movie showing a close-up view of etching process of ZIF by molten Co nanoparticles at 850 °C in Figure 3i–l. The movie is displayed at 2× speed.
